# Dendritic cell immunoreceptor 2 (DCIR2) deficiency decreases hepatic conventional dendritic cell content but not the progression of diet‐induced obesity

**DOI:** 10.1002/iid3.1024

**Published:** 2023-10-25

**Authors:** Rossella Bellini, Annalisa Moregola, Jasmine Nour, Patrizia Uboldi, Fabrizia Bonacina, Giuseppe D. Norata

**Affiliations:** ^1^ Department of Excellence of Pharmacological and Biomolecular Sciences Università degli Studi di Milano Milan Italy

**Keywords:** Clec4a4, C‐type lectin receptors, DCIR2, dendritic cells, obesity

## Abstract

**Aims:**

Inflammatory pathways and immune system dysregulation participate in the onset and progression of cardiometabolic diseases. The dendritic cell immunoreceptor 2 (DCIR2) is a C‐type lectin receptor mainly expressed by conventional type 2 dendritic cells, involved in antigen recognition and in the modulation of T cell response. Here, we investigated the effect of DCIR2 deficiency during the development of obesity.

**Methods:**

DCIR2 KO mice and the WT counterpart were fed with high‐fat diet (HFD) for 20 weeks. Weight gain, glucose and insulin tolerance were assessed, parallel to immune cell subset profiling and histological analysis.

**Results:**

After HFD feeding, DCIR2 KO mice presented altered conventional dendritic cell distribution within the liver without affecting markers of hepatic inflammation. These observations were liver restricted, since immune profile of metabolic and lymphoid organs‐namely adipose tissue, spleen and mesenteric lymph nodes‐did not show differences between the two groups. This reflected in a similar metabolic profile of DCIR2 KO compared to WT mice, characterized by comparable body weight gain as well as adipose tissues, spleen, Peyer's patches and mesenteric lymph nodes weight at sacrifice. Also, insulin response was similar in both groups.

**Conclusion:**

Our data show that DCIR2 has a redundant role in the progression of diet‐induced obesity and inflammation.

## Graphical abstract

The deficiency of DCIR2 ‐ a marker of conventional type 2 dendritic cells ‐ affects hepatic DC distribution but not lipid accumulation in liver and adipose tissues under high‐fat diet. Based on our findings, the absence of DCIR2 has a redundant role in the progression of diet‐induced obesity.

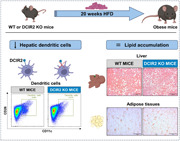

Ectopic fat accumulation is the hallmark of obesity and promotes moderate chronic inflammation, which accelerates the development of cardiovascular disease, diabetes, and cancer.^1^ Lipid deposition triggers the activation of an immune‐inflammatory response that prompts the proinflammatory activation and migration of immune cells in the adipose tissue and in the liver.^2^ In this setting, the role of dendritic cells (DCs), which act as a bridge between innate and adaptive immunity,^2^ is still poorly explored.

DCs are bone marrow‐derived cells, categorized into classical or conventional DCs (cDCs), plasmacytoid DCs (pDCs), monocyte‐derived DCs (MoDCs), and Langerhans cells (LCs). They reside in different tissues where they sense stimuli that impact to their activation, polarization, and maturation.^3^ While it has been shown that adipose tissue DCs are central players in the initiation and progression of obesity‐induced inflammation and insulin resistance,^3^ the contribution of DCs to metabolic failure of other organs, such as the liver, is less clear. Here, DCs mainly reside within the periportal and pericentral space^4^ and, although it is known that they contribute to tissue homeostasis as well as obesity‐associated immune response,^3^ their role is still controversial. The infiltration of CX3CR1^+^ MoDCs in injured liver driven by increased CX3CL1 expression, supports local inflammatory response^5^ and, accordingly, blocking CX3CR1 was shown to limit hepatic inflammation and damage.^4^ Instead, recent publications have proposed different functions of DCs according to their lipid content: while lipid‐enriched liver‐derived DCs show a proinflammatory role that supports the activation of the effector arm of the adaptive immune response, lipid‐poor DCs promote T‐cell mediated tolerance.^6^ In addition, DCs also contribute to liver fibrosis^7^ and, particularly, liver type 2 conventional DCs (cDC2) positively correlate with the progression of metabolic steatohepatitis.^4^

A peculiar characteristic of murine cDC2 is the expression of the dendritic cell immunoreceptor 2 (DCIR2), whose role—beyond being a cDC2 marker—has not been fully elucidated. Indeed, DCIR2 has been involved in the induction of the tolerogenic CD4^+^ T‐cell response in NOD mice,^8^ while its deficiency enhanced cytokine production and T‐cell priming following Toll‐like receptor (TLR)‐mediated activation in vivo^9^ but was shown to limit atherogenesis in hypercholesterolemic mice.^10^

Thus, this work aimed to investigate the impact of DCIR2 deficiency during obesity and metabolic syndrome development in high‐fat diet (HFD)‐fed mice.

First, we evaluated the impact of DCIR2 deficiency on immune cell distribution after 20 weeks of HFD feeding. Total DCs and cDC2 cells were decreased in the liver of DCIR2 KO mice, while the levels of cDC1 and MoDCs remained unchanged compared to matched controls (Figure 1A–F). Although the number of cDC2 was reduced, the expression of costimulatory molecules such as *CD40*, *CD80*, *CD86*, and the chemokine receptor *CX3CR1*, which contribute to the activation and recruitment of DCs, was similar in the two groups (Figure 1G), thus excluding differences in the activation status of immune cells in the liver. In line with these results, similar levels of CD3^+^ T lymphocytes within the liver, both CD4^+^ and CD8^+^ cells, as well as the percentage of T regulatory cells (Figure 1H–K) were reported. Further characterization of T lymphocyte polarization was performed through the profiling of transcriptional factors *Tbet*, *RORγT*, and *GATA3*, which are master regulators of T helper (Th)1, Th17, and Th2 response, respectively. This analysis showed decreased *GATA3* expression (Figure 1L), supporting the observation that cDC2 are mainly involved in the maintenance of Th2 immunity. Together, these results indicate that DCIR2 deficiency impacts cDC2 resulting in reduced Th2 cell polarization within the liver, without directly affecting the gene expression of *IL6, IL17, IL1β, TNF‐α, TGFβ*, and *IL23* as parameters of hepatic inflammation (Figure 1M).

**Figure 1:**
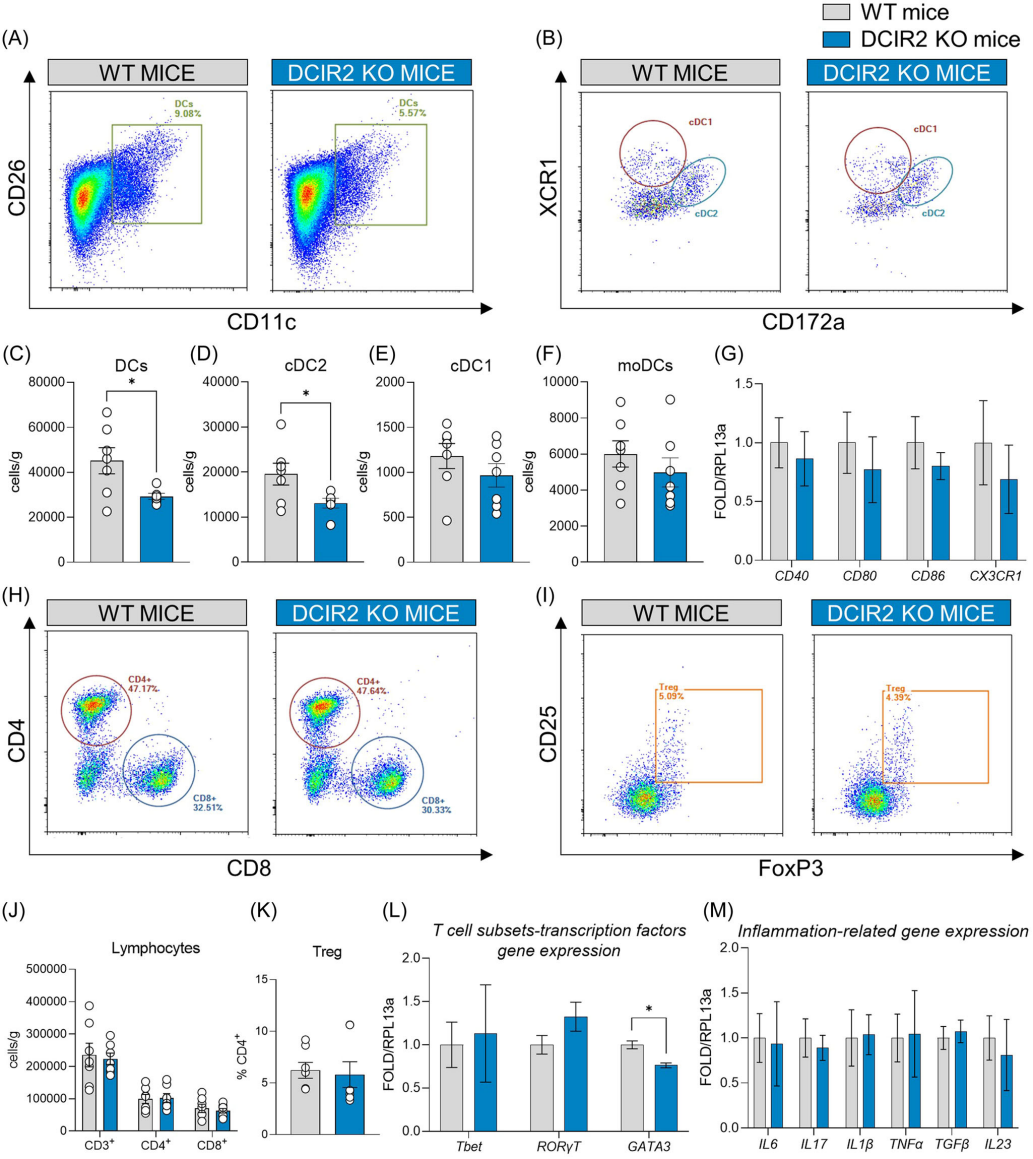
DCIR2 deficiency impacts hepatic dendritic cell distribution without affecting tissue inflammation. (A) Representative flow cytometry plots showing dendritic cells (DCs), identified as CD11c^+^ CD26^+^ cells in the liver. (B) Representative flow cytometry plots showing conventional DC subsets identified within CD11c^+^ CD26^+^ and XCR1^+^ (conventional type 1 dendritic cells, cDC1) or CD172a^+^ (conventional type 2 dendritic cells, cDC2). (C–F) Flow cytometry analysis of DCs, cDC2, cDC1, MoDCs cell count per gram of liver. (G) Hepatic gene expression of *CD40*, *CD80*, *CD86*, and *CX3CR1*. (H–I) Representative flow cytometry plots showing CD4^+^ and CD8^+^ T lymphocytes and T regulatory cells (Treg). (J) Flow cytometry analysis of CD3^+^, CD4^+^, and CD8^+^ T lymphocytes in the liver of WT and DCIR2 KO after 20 weeks of high‐fat diet (HFD); data are presented as cell count per gram of liver. (K) T regulatory cell percentage on CD4^+^ T lymphocytes. (L) Gene expression analysis of T lymphocytes transcription factors, *Tbet*, *RORγT*, and *GATA3*. (M) Gene expression analysis of inflammation‐related molecules *IL6, IL17, IL1β, TNF‐α, TGFβ, IL23*. Results are expressed as mean ± SEM. *n* = 7–4 per group. Statistical analyses were performed with unpaired *t*‐tests or multiple unpaired *t*‐test.*p < .05.

These differences were however restricted to the liver since visceral adipose tissue (Supporting Information: Figure S2A), and lymphoid organs, such as spleen, mesenteric lymph nodes (mLNs), and Peyer's patches showed a similar distribution of DCs between DCIR2 KO and WT mice (Supporting Information: Figure S2B–D), thus suggesting that the impact of DCIR2 deficiency is mainly affecting the liver.

To next explore whether these changes might have resulted in a different metabolic response of DCIR2 KO mice under high caloric intake, we investigated the effect of HFD feeding on metabolic phenotype. Body weight (Supporting Information: Figure S3A) and weight gain (Figure 2A) were similar in DCIR2 KO and WT mice, and the same was true for plasma lipid levels (Figure 2B,C). Moreover, the percentage of liver, pancreas, visceral adipose tissue (VAT), subcutaneous adipose tissue (SCAT), and brown adipose tissue (BAT) weight on body weight was similar among the animal models (Figure 2D,E). Also, the weight of the lymphoid tissues—thymus and spleen—was not different (Figure 2F). Interestingly, glycemia after overnight fasting was slightly elevated in DCIR2 KO compared to WT mice (Figure 2G), and DCIR2 KO mice were less glucose tolerant compared to WT mice (Figure 2H and Supporting Information: Figure S3B), showing a preserved insulin tolerance (Supporting Information: Figure S3C). Despite this result would be possibly explained by a decreased trend of circulating insulin levels of KO mice compared to WT observed at two timepoints of GTT (Supporting Information: Figure S3D), lipid deposition in the liver parenchyma was similar between WT and KO mouse (Figure 2I,J and Supporting Information: Figure S4A), casting for a redundant role of DCIR2 in the metabolic response.

**Figure 2:**
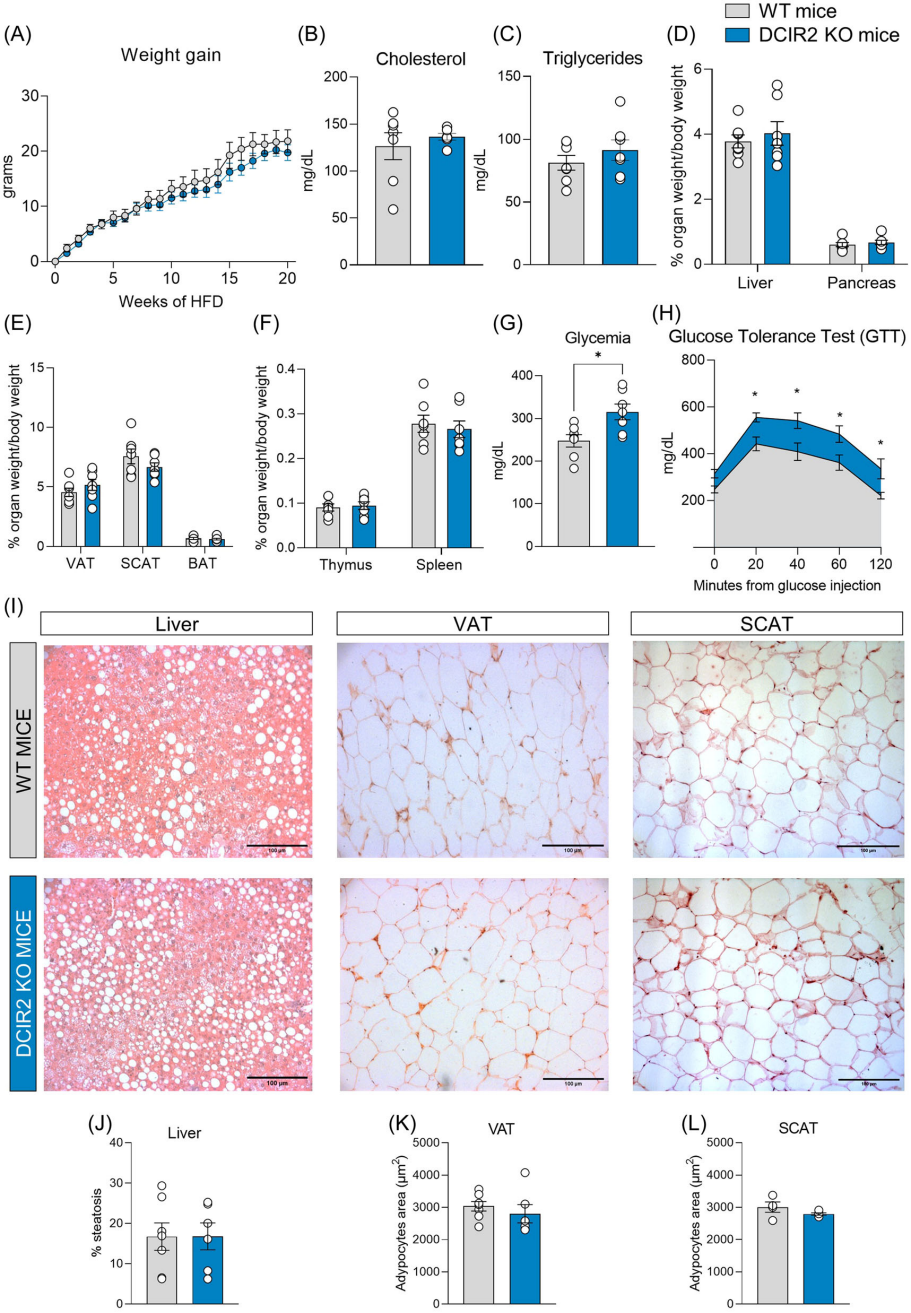
The lack of DCIR2 does not impact obesity development. (A) Body weight gain during 20 weeks of high‐fat diet (HFD) feeding of WT and DCIR2 KO mice. (B, C) Plasma cholesterol and triglyceride levels of WT and DCIR2 KO mice after 20 weeks of HFD after overnight fasting. (D–F) Liver, pancreas, VAT, SCAT, BAT, thymus, and spleen weight in WT and DCIR2 KO mice. (G) Plasma glucose levels after overnight fasting in WT and DCIR2 KO mice. (H) Plasma glucose levels in WT and KO mice following intraperitoneal glucose tolerance test (0, 20, 40, 60, and 120 min). (I) Representative images of the liver, VAT, and SCAT sections after H&E staining at ×10 magnification. (J) Percentage of hepatic steatosis. (K, L) Adipocyte area in VAT and SCAT. Results are expressed as mean ± SEM. *n* = 7 per group. Statistical analyses were performed with unpaired *t*‐test.*p < .05.

This extended also to adipose tissue, where adipocyte area was similar in both visceral and subcutaneous adipose tissue (VAT and SCAT, respectively) (Figure 2I,K,L). Similarly, the expression of markers of adipose tissue biology, such as *PPARγ*, and *adiponectin* (Supporting Information: Figure 4B) was similar in WT and KO adipose tissue. We then considered the expression of dipeptidyl peptidase‐4 (CD26), an amino peptidase that reduces the activity of incretin peptides (glucagon‐like peptide 1 and glucose‐dependent insulinotropic polypeptide) resulting in reduced insulin secretion and, even, insulin resistance.^11^ However, although our flow cytometry analysis showed a similar expression of CD26 in visceral DCs of WT and DCIR2 KO (Supporting Information: Figure 4C), we cannot exclude that DCIR2 deficiency could have been compensated by other mechanisms, thus masking its contribution to obesity‐associated inflammation. Indeed, given its role in the maintenance of immune tolerance, it is plausible that its function is redundant with other similar pathways.

In conclusion, the profiling of DCIR2 KO fed on HFD for 20 weeks indicates that DCIR2 has a redundant role in obesity and obese‐related inflammation. Indeed, despite the absence of DCIR2 impacted DC distribution and Th2 polarization within the liver, this did not affect liver steatosis or adipose tissue inflammation and lipid accumulation.

Details on material and methods are presented as supplemental material.

## AUTHOR CONTRIBUTIONS

**Rossella Bellini**: Formal analysis; investigation; validation; visualization; writing—original draft. **Annalisa Moregola**: Investigation; methodology; validation. **Jasmine Nour**: Investigation. **Patrizia Uboldi**: Investigation. **Fabrizia Bonacina**: Conceptualization; formal analysis; funding acquisition; methodology; project administration; resources; supervision; validation; visualization; writing—original draft; writing—review and editing. **Giuseppe D. Norata**: Conceptualization; funding acquisition; project administration; resources; supervision; writing—original draft; writing—review and editing.

## CONFLICT OF INTEREST STATEMENT

The authors declare no conflict of interest.

## Supporting information

Supporting information.

## Data Availability

The data supporting this study's findings are available from the corresponding author, G.D.N., upon reasonable request.
