# Supplementary material for: Dendritic cell immunoreceptor 2 (DCIR2) deficiency decreases hepatic conventional dendritic cell content but not the progression of diet‐induced obesity
Source: Immun Inflamm Dis. 2023 Oct 25;11(10):e1024. doi: 10.1002/iid3.1024 (PMC10599273; doi:10.1002/iid3.1024)
Supplement: Supplementary file 1 — Supporting information. [file IID3-11-e1024-s001.docx]

**SUPPLEMENTARY MATERIAL**

***Obesity mouse model.***

WT mice on C57BL/6J background were purchased from The Jackson Laboratory (USA) and crossed with DCIR2 KO mice (1). Animals were housed two-three per cage with free access to food and water, in a temperature (20±2°C, 50±5% relative humidity) and a 12-h light/dark cycle-controlled environment. 8-week-old WT and DCIR2 KO male littermates were randomized on a high-fat diet (HFD 45% Kcal from fat, Cat. No. D12451, Research diets Inc., New Brunswick, NJ, USA) feeding for 20 weeks with weekly monitoring of the weight. After overnight fasting, mice were sacrificed by 2% isoflurane inhalation, and the blood was collected by intracardiac puncture followed by the liver, mesenteric lymph nodes, Peyer’s patches, spleen, thymus, pancreas, visceral (VAT), subcutaneous (SCAT), and brown (BAT) adipose tissue collection. All animal procedures performed conform to the guidelines from directive 2010/63/EU of the European Parliament on the protection of animals used for scientific purposes and were approved by the Ethical Committee (Autorizzazione Ministeriale 65/2020-PR). All sections of this report adhere to the ARRIVE Guidelines; a completed ARRIVE guidelines checklist is included in Checklist S1.

***Glucose Tolerance Test, Insulin Tolerance Test, and ELISA assay.***

The glucose tolerance test (GTT) and the insulin tolerance test (ITT) were performed as already described (2). Briefly, GTT was performed after 18 weeks of HFD treatment. After overnight (12-16h) fasting, mice were weighed, and blood glucose was measured from the tail using a glucose meter (ONE-TOUCH Ultra, Lifescan, Milpitas, CA, USA). Measurements were performed before and after 20, 40, 60, and 120 minutes from intraperitoneal injection of glucose solution (10% w/v in physiological solution (NaCl 0.9%). At the end of the test, mice had *ad libitum* access to food. Before and after 60 minutes of glucose injection, 40μL of blood were collected in Eppendorf containing EDTA 0.5%. Blood samples were centrifuged for 10 min at 6000rcf at 4°C and plasma was collected and immediately stored at -20°C for ELISA assay. The concentration of insulin was measured by using an Ultrasensitive Mouse insulin ELISA assay (Mercodia, Cat n°10-1249-01) following instructions, with a plasma dilution of 1:5. Insulin Tolerance Test (ITT) was performed after 19 weeks of HFD treatment. Mice were fasted for 4h, weighed, and blood glucose was measured following the collection of a blood drop using a glucose meter. Measurements were performed before and after 20, 40, 60, and 120 minutes from intraperitoneal injection of human recombinant insulin (0.75 mU per gram of body weight, Humulin R U-100, 100 U/mL), in a physiological solution (NaCl 0.9%) with 3% of bovine serum albumin (BSA Sigma-Aldrich). At the end of the test, mice were provided with food.

***Plasma triglyceride and cholesterol quantification.***

Plasma triglyceride and cholesterol quantification were performed as previously described. (1). Briefly, after overnight fasting, blood was collected from sacrificed animals and plasma was separated by centrifugation. Plasma cholesterol and triglycerides concentrations were quantified using the Cholesterol CP KIT (ABX Pentra, HORIBA Medical) or the triglyceride CP KIT (ABX Pentra, HORIBA Medical) with a spectrophotometer at 490 nm (Bio-Rad iMark microplate reader).

***Tissue sample preparation for flow cytometry.***

Mesenteric lymph nodes (mLNs), Peyer’s patches, and spleen were passed through 70 μm cell-strainers in PBS with FBS 2% and EDTA 2mM to obtain single cell suspension, followed by antibody staining. For spleen and blood staining, erythrocytes were lysed after 5 minutes of incubation with Red Blood Cell (RBC) Lysis Buffer (eBioscience™, Cat#00-4333-57) on ice. Cells were then washed with PBS with FBS 2% and EDTA 2mM and resuspended for antibody staining. VAT single-cell suspension was obtained after collagenase digestion (as previously described (3)). The liver was smashed and passed through 100 μm cell strainers with PBS with FBS 2% and EDTA 2mM. Then, a centrifuge at 50rcf brake-off for 1 minute was performed to pellet debris. The supernatant was recovered and centrifuged at 700g high-brake for 8 minutes at RT. The pellet was then resuspended in 10 mL of 37.5% Percoll (Sigma-Aldrich), 3.75% di PBS 10x, 58.75% di RPMI 1640 (Euroclone S.p.A., Milan, Italy) and centrifuged at 850g brake off for 30 minutes at RT. The upper layer was discarded, and the recovered pellet was incubated in RBC lysis buffer for 5 minutes on ice, washed, and used for flow cytometry staining.

***Flow cytometry.***

Immunophenotyping was performed on single-cell suspensions previously obtained from the liver, mLNs, spleen, VAT, and Peyer’s patches. Cell suspensions were superficially stained with the antibody mixture for 30 minutes at 4°C. For intracellular staining of FoxP3 and other transcription factors in liver T cells, samples were previously fixed and permeabilized (Foxp3 / Transcription Factor Staining Buffer Set, Cat# 00-5523-00, eBioscience) according to manufacturer instructions. Washes were performed with either PBS with FBS 2% and EDTA 2mM for superficial staining, or Perm Wash buffer for intracellular staining, and samples were finally resuspended in PBS with FBS 2% and EDTA 2mM. Samples were acquired with LSRFortessa X-20 (BD eBioscience) and analyzed with NovoExpress software (version 1.6.0, Agilent, Santa Clara, CA, USA). Antibodies used are listed in the Supplementary Table S1.

***Quantitative real-time PCR (qRT-PCR).***

For gene expression analysis, 10mg of liver were homogenized with TissueRuptor II (Qiagen), and total RNA was extracted using Monarch^®^ Total RNA Miniprep Kit (Cat#T2010S, New England BioLabs) following manufacturer’s instructions. 50mg of VAT were homogenized with TissueRuptor II (Qiagen) and the total RNA was extracted using Qiagen RNeasy Lipid Tissue Mini Kit (Cat#74804). RNA was retro-transcribed to cDNA with iScript™ Reverse Transcription Supermix (Cat#1708841, Bio-Rad), from which 5 ng of cDNA were amplified by real-time quantitative PCR with Luna^®^ Universal qPCR Master Mix (Cat#M3003E, NEB) and CFX Connect Real-Time PCR Detection System instrument (Cat#1855201, Bio-Rad). 1 µM of each primer was used to a final volume of 15µL of reaction mix with 13µL of master MIX. The genes used are listed in the Supplementary Table S2.

The threshold cycle number (Ct) values for each reaction were calculated and gene expression was determined as 2^-ΔΔCt^ and then as a fold of difference of KO to the control group adjusted to the expression of the housekeeping *Rpl-13a*.

***Liver and VAT histology.***

After sacrifice, fresh livers, VAT and SCAT were collected and fixed overnight in PBS/formaldehyde 4% (Cat#252549, Sigma-Aldrich, St. Louis, MO, USA,) followed by paraffin-embedding. Paraffin sections of 5 µm thickness, were obtained with a Leica Biosystem microtome, collected, and stained with Hematoxylin and Eosin (H&E) (Sigma-Aldrich). Images were acquired with an optical microscope at a 10x magnification using the Axiovision Zeiss software (version 4.8, Carl Zeiss, Oberkochen, Germany). The evaluation of liver steatosis was performed using ImageJ software and the adipocyte area of VAT and SCAT using the Adiposoft Plugin, analyzing 10 sections per animal.

***Statistical analysis.***

Graphical presentations and statistical analysis were performed with Graph Pad-Prism. Results are expressed as mean per group ± SEM. The statistical analyses between the two groups were performed by unpaired parametric two-side T-test or ordinary two-way ANOVA Sidak’s multiple comparisons test with a 95% confidence interval. A *p*-value of <0.05 was considered significant.

**Supplementary Table 1**

List of conjugated antibodies used for flow cytometry analysis.

***Liver, VAT, Spleen, mLNs, Peyer’s patches staining.***

| **Antibody** | **Clone** | **Cat#** | **Company** |
| --- | --- | --- | --- |
| LIVE/DEAD™ Fixable Aqua Dead Cell Stain Kit | - | L34957 | Invitrogen™ |
| Anti-mouse CD45 BUV563 | 30-F11 | 612924 | BD Biosciences |
| Anti-mouse CD64 PE/DAZZLE | X54-5/7.1 | 139319 | Biolegend |
| Anti-mouse F4/80 AF647 | T45-2342 | 565853 | BD Biosciences |
| Anti-mouse Lineage PerCP Cy5.5 |  | 561317 | BD Biosciences |
| Anti-mouse I-A-I-E BV650 | M5/114.15.2 | 563415 | BD Biosciences |
| Anti-mouse CD26 BV711 | H194-112 | 740678 | BD Biosciences |
| Anti-mouse CD11c BV786 | HL3 | 563735 | BD Biosciences |
| Anti-mouse XCR1 FITC | ZET | 148209 | Biolegend |
| Anti-mouse CD172a BUV395 | P84 | 740282 | BD Biosciences |

**Supplementary Table 2**

List of forward and reverse primers used for quantitative Real-Time PCR (qRT-PCR) on liver and VAT

| **Gene** | **Forward sequence** | **Reverse sequence** |
| --- | --- | --- |
| *Rpl-13a* | 5'-GCGCCTCAAGGTGTTGGAT-3' | 5'-GAGCAGCAGGGACCACCAT-3' |
| *CD11c* | 5'-ACACTGAGTGATGCCACTGT-3' | 5'-AGGTCACCTAGTTGGGTCTT-3' |
| *CD40* | 5'-CCTGCGATGGTGTCTTTGC-3' | 5'-AGATGGACCGCTGTCAACAAG-3' |
| *CD80* | 5'-AGGAAGCCTACGGGCAAGTT-3' | 5'-GGCTCAGCCTTTCCACTTCA-3' |
| *CD86* | 5'-GGGCCGCACGAGCTTT -3' | 5'-CCCATGTCCTTGATCTGAACATT-3' |
| *CX3CR1* | 5'-TCAGCATCGACCGGTACCTT -3' | 5'-CTGCACTGTCCGGTTGTTCAT-3' |
| *Tbet* | 5'-TGAGAGCCCCGAGCTCTTC -3' | 5’-CCCCGCTTCCTCTCCAA -3' |
| *RORgT* | 5'-CAGCCAACATGTGGAAAAGCT -3' | 5'-GGGAAGGCGGCTTGGA -3' |
| *Gata3* | 5'-GAACCGGCCCCTTATCAAG -3' | 5'-CAGGATGTCCCTGCTCTCTT-3' |
| *Il6* | 5'-CTGCAAGAGACTTCCATCCAGTT-3' | 5'-AGGGAAGGCCGTGGTTGT-3' |
| *Il17* | 5’-CAAACACTGAGGCCAAGGAC-3’ | 5’-TCTTCATTGCGGTGGAGAGT-3’ |
| *Il1β* | 5'-GGTGTGTGACGTTCCCATTAGA-3' | 5'-CAGCACGAGGCTTTTTTGTTG-3' |
| *TNFα* | 5'-CTCCTCACCCACACCATC-3' | 5'-GAAGACCCTCCCAGATAG-3' |
| *TGFβ* | 5'-TCGACATGGAGCTGGTGAAA-3' | 5'-GAGCCTTAGTTTGGACAGGATCTGGCCAC-3' |
| *Il23* | 5'-CCGTTCCAAGATCCTTCGAA-3' | 5'-CAAAGACCCGGGCAGCTA -3' |
| *Adiponectin* | 5'-AATTCTGAAGCCATTGTCTCC-3' | 5'-AGTTATTGTCTCCTGAGAGAATG -3' |
| *PPARγ* | 5'-CCATTCTGGCCCACCAACT-3' | 5'-TGCGAGTGGTCTTCCATCAC-3' |

**References**

1. Bellini R, Moregola A, Nour J, Rombouts Y, Neyrolles O, Uboldi P, Bonacina F, Norata GD. Dendritic cell marker Clec4a4 deficiency limits atherosclerosis progression. *Atheroscler Plus* (2023) **51**:8–12. doi: 10.1016/J.ATHPLU.2022.12.001

2. Da Dalt L, Ruscica M, Bonacina F, Balzarotti G, Dhyani A, Di Cairano E, Baragetti A, Arnaboldi L, De Metrio S, Pellegatta F, et al. PCSK9 deficiency reduces insulin secretion and promotes glucose intolerance: the role of the low-density lipoprotein receptor. *Eur Heart J* (2020) **40**:357–368. doi: 10.1093/EURHEARTJ/EHY357

3. Macchi C, Moregola A, Greco MF, Svecla M, Bonacina F, Dhup S, Dadhich RK, Audano M, Sonveaux P, Mauro C, et al. Monocarboxylate transporter 1 deficiency impacts CD8+ T lymphocytes proliferation and recruitment to adipose tissue during obesity. iScience (2022) 25: doi: 10.1016/J.ISCI.2022.104435

**Supplementary Figures*
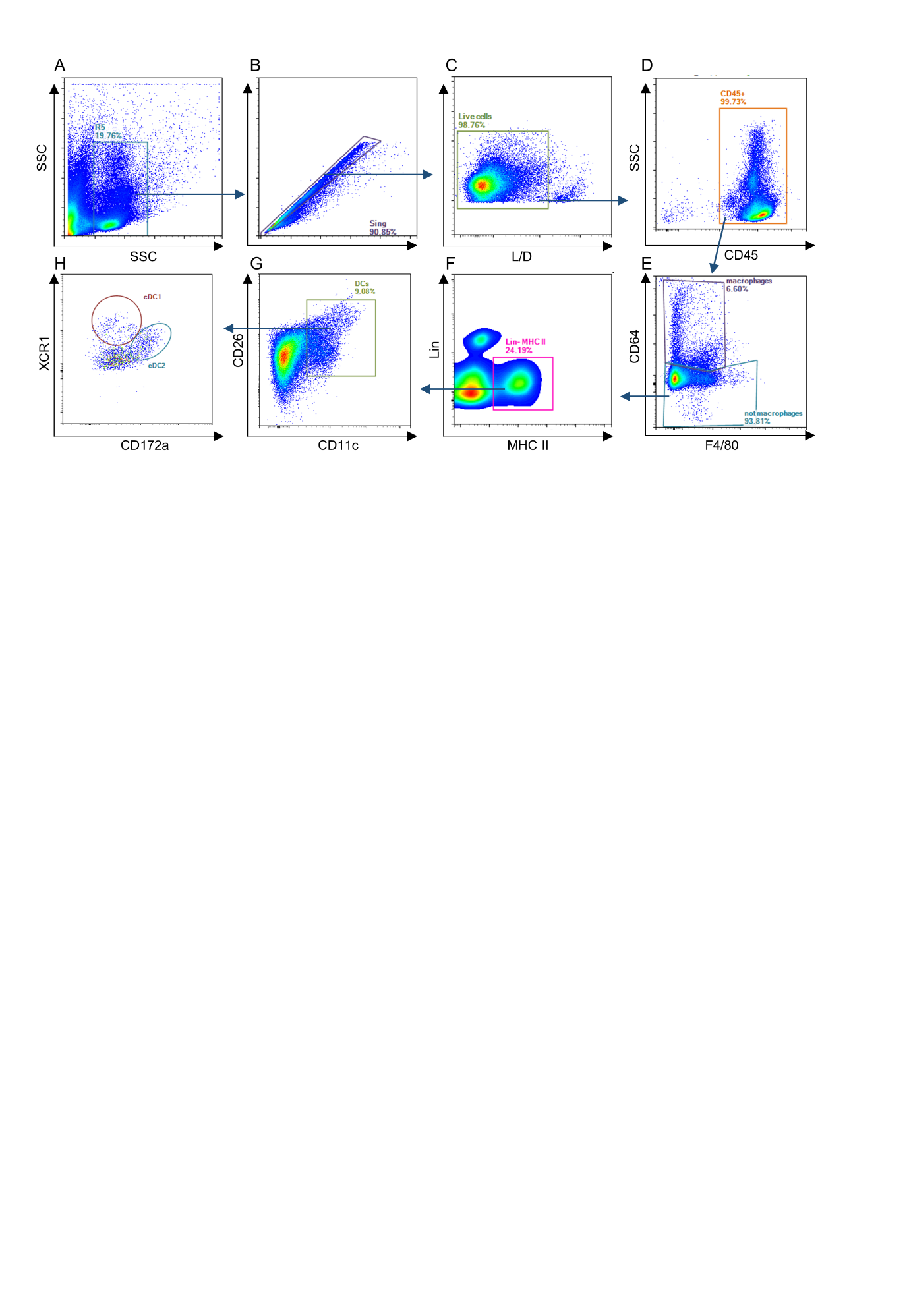
***

***Suppl. Fig.1 Gating strategy for liver, VAT, Spleen, mLNs, and Peyer’s patches’ dendritic cell immunophenotyping.***

Representative gating strategy used for flow cytometry analyses for identifying main immune populations in liver, VAT, spleen, mLNs, and Peyer’s patches. (A-D) Cells were gated on singlets and live leukocytes were identified as CD45^+^. (E-G) Next, macrophages were excluded based on CD64^+^ positivity and gradual positivity for F4/80; from the remaining cells, DCs were isolated based first on their positivity for MHC II, from which double-positive CD26^+^ and CD11c^+^ DCs were selected. H) Dendritic cells were then divided into type 1 conventional dendritic cells (cDC1: CD172^-^ XCR1^+^) and type 2 conventional dendritic cells (cDC2: CD172a^+^ XCR1^-^).


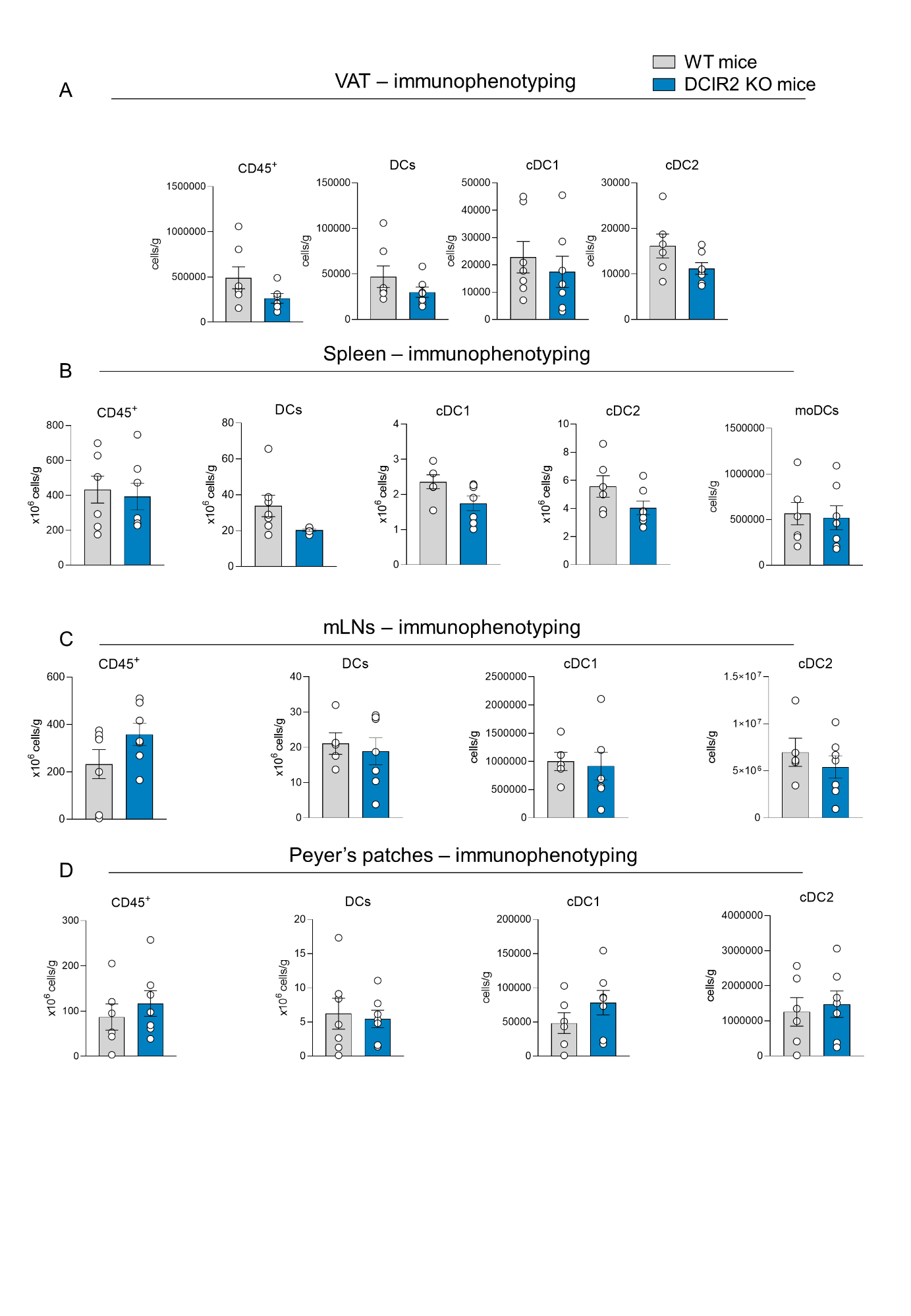
***Suppl. Fig.2 The lack of DCIR2 did not impact dendritic cell distribution in VAT, Spleen, mLNs, and Peyer’s patches.***

Panels reporting immune populations observed by flow cytometry in VAT, spleen, mLNs, and Peyer’s patches of WT and KO male mice on HFD. A) Number of total circulating CD45^+^ leukocytes, dendritic cells, cDC1, and cDC2 within the VAT, expressed as the number of cells per gram of tissue. B) Number of total circulating CD45^+^ leukocytes, dendritic cells, cDC1, cDC2, and MoDCs within the spleen expressed as number of cells per gram of tissue. C) Number of total circulating CD45^+^ leukocytes, dendritic cells, then divided into cDC1 and cDC2 within the mLNs and D) within the Peyer’s patches expressed as the number of cells per gram of tissue. Results are expressed as mean ± SEM. n=7 per group. Statistical analyses were performed with unpaired t-tests.

***
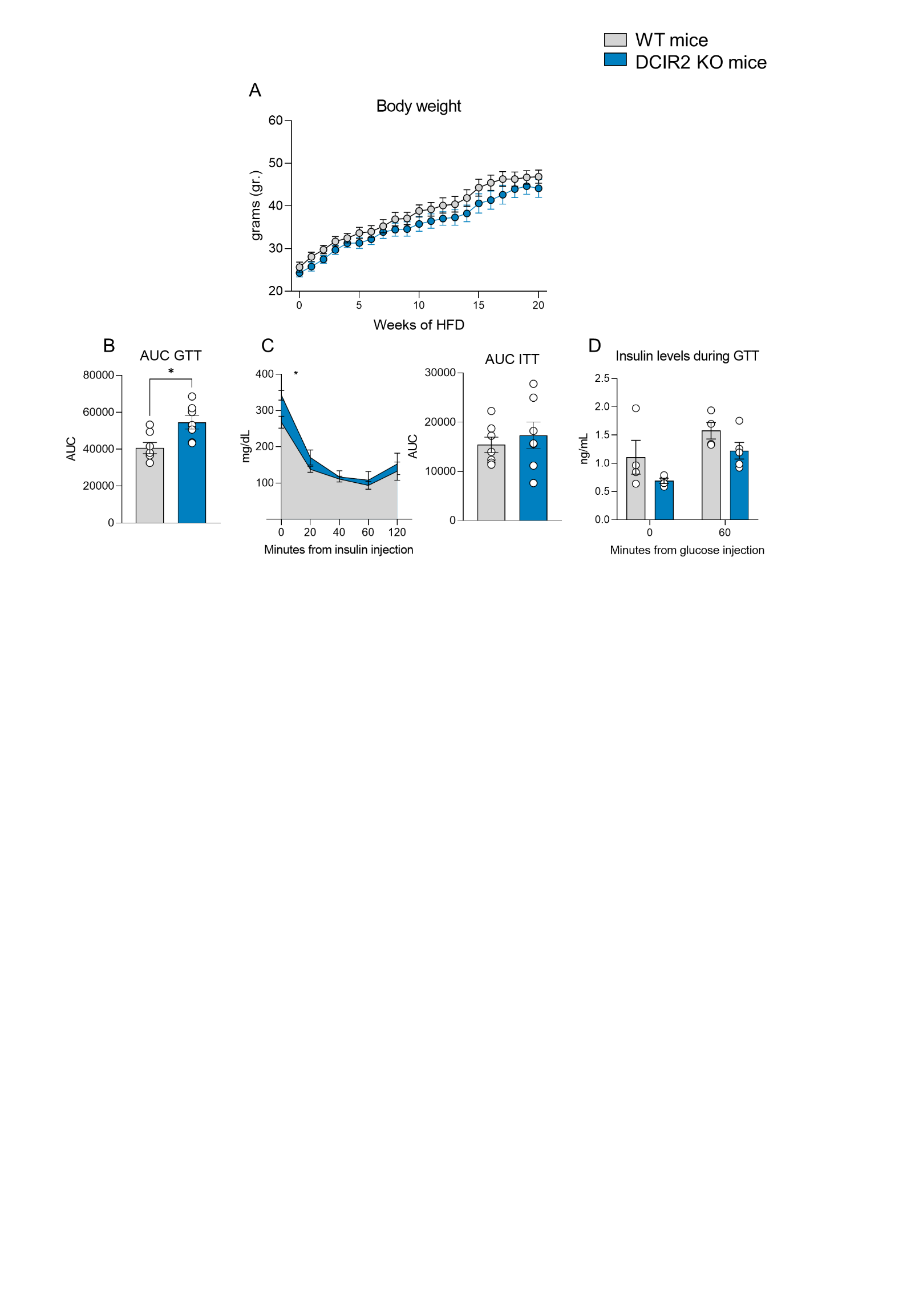
Suppl. Fig.3 DCIR2 KO mice showed the same body weight with less glucose tolerance and preserved insulin tolerance.***

A) Total body weight of WT and KO mice during 20 weeks of HFD. B) Area under the curve (AUC) of glucose tolerance test performed at 18 weeks HFD fed WT and KO mice. C) Insulin tolerance test performed on 19 weeks HFD fed WT and KO mice expressed as mg/dL and the related AUC. D) Plasma insulin levels after overnight fasting and after 60 minutes from glucose injection, expressed as ng/mL. Results are expressed as mean ± SEM. n=7 per group or n=4 per group in insulin levels analysis. Statistical analyses were performed with unpaired t-test or ordinary two-way ANOVA Sidak’s multiple comparisons test.**p*<0.05.

**
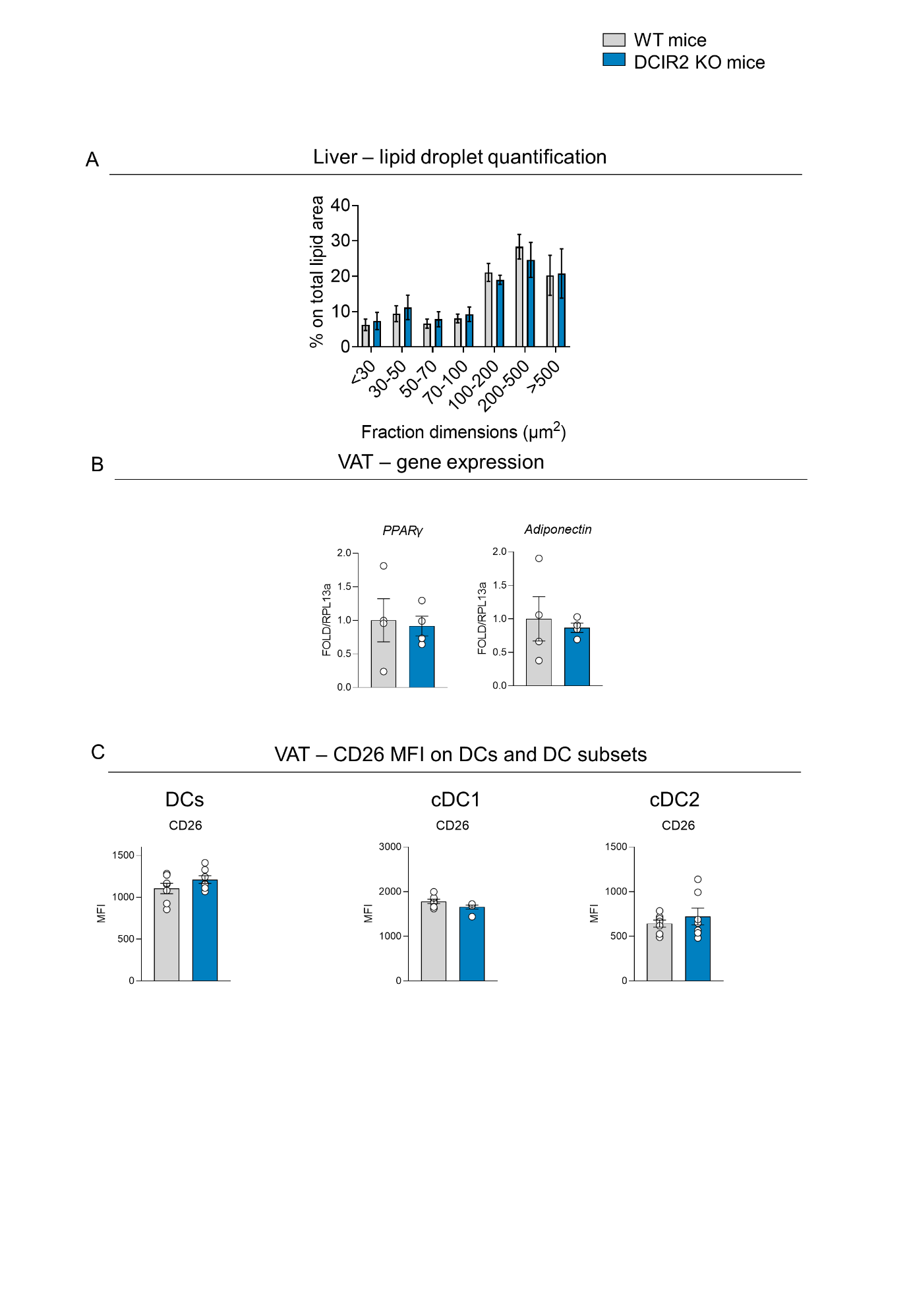
**

***Suppl. Fig.4 The lack of DCIR2 did not affect hepatic lipid deposition and adipose tissue phenotype.***

A) Distribution of lipid droplets within the liver, based on their dimensions. B) Gene expression analysis of *adiponectin* and *PPARγ* within visceral adipose tissue (VAT) expressed as fold on *RPL13a*. C) Flow cytometry analysis of CD26 mean fluorescence intensity (MFI) gated on adipose tissue DCs (ATDCs), and on cDC1 and cDC2. Results are expressed as mean ± SEM. n=7 per group or n=4 per group in gene expression analysis. Statistical analyses were performed with unpaired t-tests.
